# Female ornamentation and the fecundity trade‐off in a sex‐role reversed pipefish

**DOI:** 10.1002/ece3.4459

**Published:** 2018-08-29

**Authors:** Kenyon B. Mobley, John R. Morrongiello, Matthew Warr, Dianne J. Bray, Bob B. M. Wong

**Affiliations:** ^1^ Department of Evolutionary Ecology Max Planck Institute for Evolutionary Biology Plön Germany; ^2^ School of BioSciences University of Melbourne Melbourne Victoria Australia; ^3^ School of Biological Sciences Monash University Melbourne Victoria Australia; ^4^ Vertebrate Zoology Museum Victoria Melbourne Victoria Australia

**Keywords:** allometry, cost of reproduction, female competition, honest signaling, mate choice, sexual selection

## Abstract

Sexual ornaments found only in females are a rare occurrence in nature. One explanation for this is that female ornaments are costly to produce and maintain and, therefore, females must trade‐off resources related to reproduction to promote ornament expression. Here, we investigate whether a trade‐off exists between female ornamentation and fecundity in the sex‐role reversed, wide‐bodied pipefish, *Stigmatopora nigra*. We measured two components of the disk‐shaped, ventral‐striped female ornament, body width, and stripe thickness. After controlling for the influence of body size, we found no evidence of a cost of belly width or stripe thickness on female fecundity. Rather, females that have larger ornaments have higher fecundity and thus accurately advertise their reproductive value to males without incurring a cost to fecundity. We also investigated the relationship between female body size and egg size and found that larger females suffer a slight decrease in egg size and fecundity, although this decrease was independent of female ornamentation. More broadly, considered in light of similar findings in other taxa, lack of an apparent fecundity cost of ornamentation in female pipefish underscores the need to revisit theoretical assumptions concerning the evolution of female ornamentation.

## 1 INTRODUCTION

In many species, males are the more competitive sex and are adorned with elaborate ornaments that are used as visual signals to attract potential mates (Andersson, 1994). Male ornaments are thought to arise through sexual selection via female mate choice with more ornamented males favored by choosy females (Andersson, 1994; Hill, 2014). Such preferences are generally ascribed to the high fitness costs of bearing such ornaments (Grafen, 1990; Hill, 2014; Walther & Clayton, 2005; Zahavi, 1975, 1977). Specifically, it has traditionally been assumed that only high quality individuals should be able to bear the cost of maintaining the most elaborate ornaments, causing the degree of ornament elaboration to function as an honest indicator of an individual's quality (Grafen, 1990; Kodric‐Brown & Brown, 1984; Nur & Hasson, 1984; Walther & Clayton, 2005; Zahavi, 1977). However, according to more recent theoretical models, costs alone may not be sufficient to explain the persistence of exaggerated ornaments in nature (Tazzyman, Iwasa, & Pomiankowski, 2013, 2014).

In contrast to what is known about male sexual ornaments, far less is known about the evolution of female ornaments (Clutton‐Brock, 2007, 2009). In some rare instances, females have evolved elaborate ornamentation without a comparable ornament in males. Female‐specific ornaments have evolved in diverse taxa, such as insects (Bussiere, Gwynne, & Brooks, 2008; Hopkins, Baudry, Candolin, & Kaitala, 2015; LeBas, Hockham, & Ritchie, 2003; Takahashi & Watanabe, 2011; Wheeler, Gwynne, & Bussière, 2012), crabs (Baldwin & Johnsen, 2012), fishes (Amundsen & Forsgren, 2001; Rosenqvist & Berglund, 2011), reptiles (LeBas & Marshall, 2000; Weiss, 2006), birds (Amundsen, 2000b; Gladbach, Gladbach, Kempenaers, & Quillfeldt, 2010; Roulin, Ducrest, Balloux, Dijkstra, & Riols, 2003), and mammals (Huchard et al., 2009). Although it is generally believed that female‐specific ornamentation evolves similarly to male ornamentation through the process of mate choice and sexual selection (Amundsen, 2000a, 2000b; Clutton‐Brock, 2007, 2009), females may also putatively evolve ornaments via social interactions including signaling dominance or social selection (i.e., competition for resources, including mates) (LeBas, 2006; Lyon & Montgomerie, 2012; Tobias, Montgomerie, & Lyon, 2012; West‐Eberhard, 1979).

One explanation for its rarity is that female ornamentation is costly, particularly with respect to fecundity (Chenoweth, Doughty, & Kokko, 2006; Clutton‐Brock, 2007, 2009; Fitzpatrick, Berglund, & Rosenqvist, 1995). In general, female investment in reproduction is greater than in males, and female quality is based on her fecundity, the quality of her eggs, and/or parental care investment (Clutton‐Brock, 2009; Trivers, 1972). Female ornaments are therefore not favored to evolve if their production is costly in terms of future investment into offspring (Fitzpatrick et al., 1995). Due to a general positive body size‐fecundity relationship in many species (e.g., Barneche, Robertson, White, & Marshall, 2018), larger females may represent a higher reproductive value to prospective mates and perhaps additional ornaments may be unnecessary (Hopkins et al., 2015). However, additional ornaments may serve to amplify information about her quality to choosy males, particularly if ornaments accentuate her body size and, hence, her fecundity (Rosenqvist & Berglund, 2011). Female ornaments may also be used as a signal in female–female competition where females compete for high quality males that provide direct and indirect genetic benefits to offspring (Bernet, Rosenqvist, & Berglund, 1998; Rosvall, 2011).

If ornament expression is an accurate indicator of fecundity, then the rate of increase of the relationship between ornament expression and fecundity should be equal (Simmons & Emlen, 2008). Deviations from a positive linear relationship should indicate a trade‐off between ornament expression and fecundity. If costs increase with ornament expression, individuals displaying large ornaments would suffer reduced fecundity. Alternatively, if costs decrease with ornament expression such that smaller individuals are saddled with a higher cost to producing the ornament, or if producing larger ornaments are cheaper in larger individuals, we would expect to see proportionately larger ornaments in larger individuals.

To date, only a handful of studies have investigated the ornament‐fecundity trade‐off in species that possess female ornaments, and evidence for such a trade‐off is generally lacking. For example, a study on the dance fly, *Rhamphomyia tarsata*, found a linear relationship in fecundity and the length of pinnate scales with female body size (LeBas et al., 2003). Similarly, studies on female ornamentation in the striped plateau lizard, *Sceloporus virgatus*, have revealed a positive relationship between ornaments, fecundity, and offspring quality (Weiss, 2006; Weiss, Kennedy, & Bernhard, 2009). In horned beetles, *Onthophagus sagittarius*, female body size was the most reliable predictor of maternal quality, yet developing relatively large horns did not impart a cost to fecundity (Simmons & Emlen, 2008). In the upland goose, *Chloephaga picta leucoptera*, female‐specific coloration was related to clutch and egg volumes (Gladbach et al., 2010), while in glow worms, *Lampyris noctiluca*, the intensity of the glow emitted by females was positively associated with body size and fecundity (Hopkins et al., 2015). In at least one case, the eider duck, *Somateria mollissima,* female‐specific plumage was unrelated to clutch size or the phenotypic quality of females (Lehikoinen, Jaatinen, & Öst, 2010). Thus, despite the theoretical costs of female ornamentation on fecundity, little empirical evidence exists to support such a scenario.

Members of the family Syngnathidae (pipefish, seahorses, and seadragons) offer an outstanding opportunity to investigate the evolution of female ornamentation because a remarkable diversity of female ornaments has evolved in several lineages, ranging from temporary courtship ornaments to extreme sexual dimorphism, brilliant permanent markings and flashy displays (Dawson, 1985; Kuiter, 2009; Rosenqvist & Berglund, 2011). Female ornaments ostensibly evolved in syngnathids because of the unique reproductive mode of this group: male pregnancy. Males provide all parental care and species with enclosed brood pouches provide protection, osmoregulation, and nutrition to developing embryos (Haresign & Shumway, 1981; Kvarnemo, Mobley, Partridge, Jones, & Ahnesjö, 2011; Partridge, Shardo, & Boettcher, 2007; Ripley & Foran, 2006, 2009). In most syngnathid species, male pregnancy decreases the rate at which males can mate but not females, thereby increasing competition between females for access to mating opportunities (Berglund, Rosenqvist, & Svensson, 1986a; Kvarnemo & Ahnesjö, 1996). Female mate‐limitation creates a biased operational sex ratio and results in sex‐role reversal where sexual selection acts more strongly on females than on males (Berglund, Rosenqvist, & Svensson, 1986b; Jones, Rosenqvist, Berglund, Arnold, & Avise, 2000; Vincent, Ahnesjo, Berglund, & Rosenqvist, 1992). Consistent with sexual selection theory, the genetic mating system across syngnathid species predicts the strength of selection for exaggerated ornamentation, that is, females from highly polyandrous species have the most striking ornaments (Jones & Avise, 2001; Rosenqvist & Berglund, 2011).

**Figure 1:**
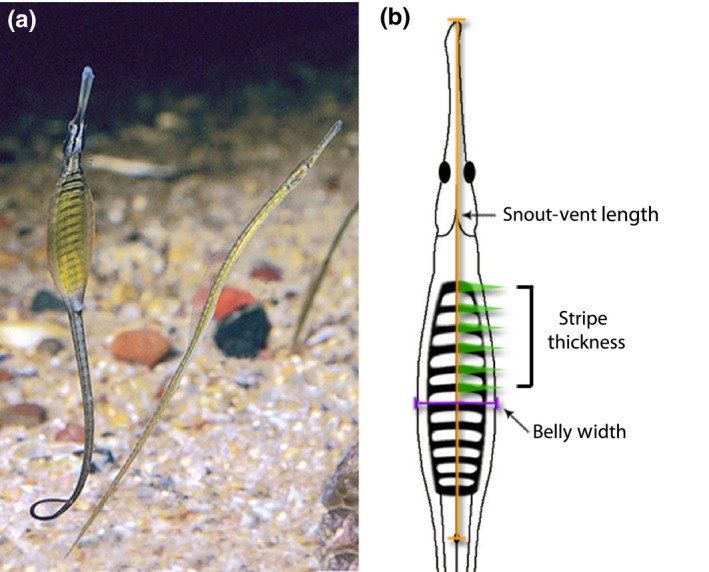
(a) Female (left) and male (right) *Stigmatopora nigra*. The female is displaying her striped belly ornament to the male. (b) Schematic diagram of measurements used in this study. Snout‐vent length is estimated from ventral photographs from the tip of the rostrum to the anal pore. Stripe thickness is the mean width of the first six dark stripes. Belly width is calculated as the widest part of the body. Photography © Rudie Kuiter, used with permission

This study aimed to investigate the trade‐off between female ornamentation and fecundity in the wide body pipefish, *Stigmatopora nigra*, Kaup 1856. This species is ideal because females possess an exaggerated ornament that females display to males during courtship. The ornament is a large dorsoventerally flattened, disc‐shaped belly with alternating light and dark ventral stripes (Figure 1). The width of the ornament and stripes can be measured directly, and fecundity can be obtained by counting mature ova in the ovaries of females. Accordingly, we explored the relationship between female fecundity and the size and stripe pattern of the female ornament. We also investigated the relationship between mean egg size and ornament expression. We predicted that if there is no fecundity cost to ornament expression, then a positive linear relationship between ornament expression and fecundity or egg size should be observed.

## 2 MATERIAL AND METHODS

### 2.1 Study species

The wide body pipefish, *S. nigra* (Kaup, 1856), occurs in bays, estuaries, and shallow coastal waters of southern Australia and New Zealand (Dawson, 1985). Wide body pipefish breed throughout the year in shallow seagrass beds and their abundance and the proportion of pregnant males reach their peak in September–January (Duque‐Portugal, 1989). Females possess a wide, laterally compressed body, a darkly pigmented dorsum and a striped, ventral ornament that is displayed to males during courtship (Figure 1a). Occasionally, females have an additional fleshy fold on the lateral edges of the ornament (Dawson, 1985). Males do not possess a wide body and lack stripes on their brood pouch (Figure 1a). Males have a semi‐inverted pouch enclosure and care for offspring until parturition (Dawson, 1985; Wilson, Ahnesjö, Vincent, & Meyer, 2003). The mating system of the species is unknown due to lack of molecular parentage studies conducted on this species. However, because of the strong sexual dimorphism and possession of a female ornament, the species is most likely polyandrous where males mate with a single female while females can mate with multiple males (Jones & Avise, 2001). This species is also putatively sex‐role reversed with respect to sexual selection (i.e., sexual selection acting more strongly on females than males) similar to other pipefish species that display female ornaments (Berglund et al., 1986b; Jones et al., 2000).

### 2.2 Sample collections

Adult *S. nigra* used in the study were museum specimens collected either using drop or seine nets at Grassy Point in Port Phillip Bay on the Bellarine Peninsula, Victoria, Australia (38°07′ S, 144°41′ E). Specimens were sampled across multiple years (1997, 1999, 2005, and 2006) during September–January as part of a series of unrelated studies (Jenkins & Hamer, 2001; Jenkins, Walker‐Smith, & Hamer, 2002; MacReadie, Hindell, Jenkins, Connolly, & Keough, 2009). These fish were euthanized immediately after capture using either a 99% ethanol solution or benzocaine before being preserved in 70% ethanol and stored in the collection at Museum Victoria.

### 2.3 Morphological measurements

Female pipefishes were photographed using a Nikon D80 digital SLR camera for morphological measurements. Females were placed on a foam board covered by a sheet of laminated paper with 1‐mm grid lines for scale. The females were then pinned down flat with their ventral side exposed, as this allowed for accurate measures of both body size and ornamental traits. Measurements were then taken from the photographs using the image analysis software ImageJ (http://rsb.info.nih.gov/ij/). Because 19% of females and 24% of males had broken tails, we used snout‐vent length (SVL, tip of rostrum to anal pore, Figure 1b) as a measure of body size as opposed to total length (TL, tip of rostrum to tip of tail). Snout‐vent length was highly correlated to total length in both sexes (female: *r* = 0.944, *t*_1,102_ = 28.90, *p* < 0.0001; male: *r* = 0.938, *t*_1,54_ = 19.88, *p* < 0.0001). We obtained two different measures of female ornamentation: belly width and stripe thickness. Belly width was measured from the widest point perpendicular to SVL to ensure uniformity of measurements (Figure 1b). We also measured belly width in males. For stripe thickness, the width of dark stripes at the midpoint of the SVL axis was measured (Figure 1b). While preservation caused fading of the dark stripes on many females, the first six stripes were visible for the majority of the females. Therefore, the mean thickness of the first six stripes was used as the measurement for stripe thickness.

### 2.4 Dissections and egg size measures

After photography, females were placed in a petri dish and submerged in water to prevent desiccation. Ovaries were dissected from females and eggs gently separated from ovarian tissue using tweezers, enumerated to estimate fecundity, and the egg diameters measured under 40× magnification using a 0.1‐mm graticule. Due to the ovoid shape of most eggs, two perpendicular measures of diameter were taken and averaged in order to estimate mean egg size.

Female ovaries contained both immature and mature eggs, although preservation made it difficult to identify the two. Counting immature eggs would overestimate a female's current fecundity, or potential clutch size, and thus eggs available to a male during mating. Therefore, we developed a method to estimate which eggs were mature in female ovaries, and hence her fecundity using the size range of newly laid eggs (without eyespots) located within the brood pouch of ethanol‐preserved males. Eggs from each male's brood pouch were dissected, enumerated for an estimate of male reproductive success and the diameters measured under 40× magnification using a 0.1‐mm graticule. We assumed that each male has eggs from just one female as developing embryos within male brood pouches were uniformly distributed throughout the pouch and at similar developmental stages. We then used linear regression to find the relationship, across males, between the minimum and maximum egg sizes in a pouch. A significant positive relationship was found between the minimum and maximum egg size found in a male's brood pouch (*r* = 0.518, *t*_1,57_ = 4.57, *p* < 0.0001). The maximum egg diameter was found to correlate to 0.613 × the minimum egg diameter found in a male's pouch + 0.587. We applied this equation to predict the smallest mature egg size of a female, dependent on the largest egg size found in her ovary. We calculated female fecundity as the sum of all eggs within a female's ovaries that were considered mature using this method.

### 2.5 Statistical analysis

We analyzed data from 104 females and 59 males for which all metrics were available (SVL, fecundity, egg size, belly width and stripe thickness in females; egg number and egg size in males, Table 1). Basic statistics were calculated in R (R Core Team, 2017). Variables were tested for normality and equal variances (Levene) and transformed, if necessary, to satisfy assumptions. Means are reported ± standard error of the mean.

**Table 1:** The number (*n*) of male and female *Stigmatopora nigra*, mean snout‐vent length (SVL), mean number of mature eggs in female ovaries (i.e., fecundity), mean number of eggs in male brood pouch, mean egg size, mean belly width and mean stripe thickness of females

	*n*	SVL (cm)	Number of eggs	Egg size (mm)	Belly width (mm)	Stripe thickness (mm)
Female	104	4.47 ± 0.06	32.9 ± 1.4	0.92 ± 0.01	4.53 ± 0.10	0.76 ± 0.01
Male	59	3.61 ± 0.08	32.7 ± 1.9	0.95 ± 0.01	2.15 ± 0.05	*–*

*Note*

All means are reported ± one standard error of the mean.

Based on SVL measurements of sexually mature adults, we found a unimodal normal distribution of male (Shapiro–Wilk *W* test: *W* = 0.9782, *p* = 0.3672) and female body sizes (Shapiro–Wilk *W* test: *W* = 0.9839, *p* = 0.2397) suggesting that *S. nigra* only live for 1 year. A unimodal distribution is corroborated with yearly sampling of this species (Duque‐Portugal, 1989) and similar to other temperate pipefishes (Braga Goncalves, Cornetti, Couperus, van Damme, & Mobley, 2017; Mobley, Small, Jue, & Jones, 2010). Larger females had proportionately larger ornaments (SVL vs. belly width: *r* = 0.824, *t*_1,102_ = 14.69, *p* < 0.0001; SVL vs. mean stripe thickness: *r* = 0.768, *t*_1,102_ = 12.11, *p* < 0.0001). Therefore, ornament traits were standardized for female body sizes using the raw residuals from ordinary least squares regressions of ornament ~ SVL.

To investigate the relationship between female ornaments and fitness correlates, we modeled female fecundity and egg size as a function of female size‐adjusted ornamentation and SVL using linear mixed‐effects models fitted in the lme4 package V 1.1‐17 in R (Bates, Mächler, Bolker, & Walker, 2015). We included a random intercept for sample year to account for potential among‐year differences in reproductive investment driven by unmeasured environmental conditions. We compared a series of increasingly complex models (fitted with maximum likelihood) that included linear and quadratic terms for ornamentation and SVL. We then used this modeling framework to explore the relationship between fecundity and egg size, accounting for SVL. We used Akaike Information Criterion corrected for small sample size (AICc, Burnham & Anderson, 2002) to select the best fit model. Models with ΔAICc < 2 were considered to have similar support. Fecundity data were natural log‐transformed to satisfy model assumptions and predictor variables were mean centered to facilitate interpretation of polynomial terms. Parameter estimates and 95% credible intervals were derived from the posterior distribution of the fixed effects in the best models (fitted with restricted maximum likelihood, REML) using 1,000 model simulations generated by the arm package for R.

## 3 RESULTS

Sexual dimorphism is apparent in this species: Females were larger and have bellies that were twice the width of males, on average (analysis of variance [ANOVA] SVL: *F*_1,161_ = 82.6, *p* < 0.0001; ANOVA belly width: *F*_1,161_ = 440.8, *p* < 0.0001; Table 1). Females varied widely in fecundity (14–89 eggs), belly width (2.2–6.7 mm), and stripe thickness (0.38–1.1 mm). There was a significant positive relationship between the two ornamental traits, belly width, and stripe thickness, when accounting for body size (analysis of covariance [ANCOVA] stripe thickness: *F*_1,101_ = 32.39, *p* < 0.0001; SVL: *F*_1,101_ = 40.79; *p* < 0.0001, no interaction between stripe thickness and SVL). Male brood pouches contained between 1 and 76 eggs (mean number of eggs: 32.7, Table 1).

**Figure 2:**
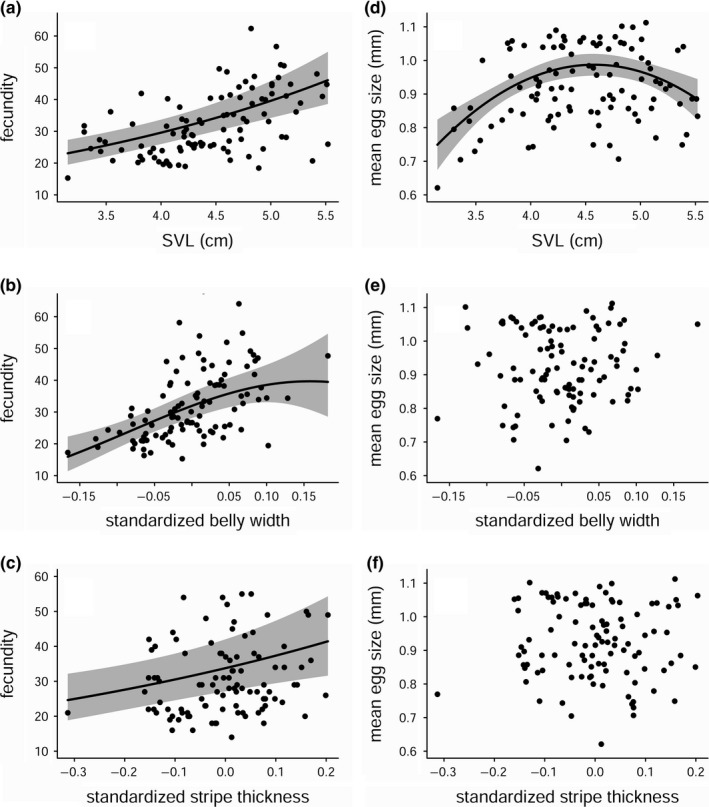
Predicted relationship (gray areas are ±95% CI) between (a) fecundity and snout‐vent length (SVL); (b) fecundity and standardized belly width (see Methods); (c) fecundity and standardized mean stripe thickness (see Methods); (d) mean egg size and SVL; (e) mean egg size and standardized belly width; (f) mean egg size and standardized stripe thickness. Points in (a) and (b) represent partial effects from multiple mixed model regression (other covariates are held at mean values). Points in c–f are observations

The best fecundity model predicted by morphological traits included linear and quadratic terms for standardized belly width and a linear term for SVL (Table 2). A model with similar support (ΔAICc = 0.7) included just linear terms for standardized belly width and SVL. Larger females were more fecund (*β*_(SVL)_ = 0.297 [95% CI 0.195–0.395], Figure 2a). Females with relatively larger belly widths also had higher fecundity (*β*_(stand.BW)_ = 2.602 [95% CI 1.703–3.617], *β*_(stand.BW2)_ = −8.518 [95% CI −18.266–1.158], Figure 2b). There was little support for the positive linear relationship between fecundity and standardized stripe thickness (ΔAICc to best Fecundity model = 34.1; Figure 2c). The best egg size model included linear and quadratic terms for SVL (Table 2). Medium sized females had the largest eggs (*β*_(SVL)_ = 1.066 [95% CI 0.597–1.498], *β*_(SVL2)_ = −0.116 [95% CI −0.165 to −0.063], Figure 2d). There was no relationship between standardized belly width or stripe thickness and egg size (Figure 2e,f).

**Table 2:** Results of AICc‐based model selection for female fecundity and mean egg size

Model	*k*	Fecundity ∆AICc	Egg size ∆AICc
Null	3	41.9	11.3
SVL	4	26.9	8.2
SVL + SVL^2^	5	29.0	**0.0**
Stand. BW	4	26.8	12.6
Stand. BW + stand. BW^2^	5	28.5	13.0
Stand. BW + SVL	5	0.7	8.9
Stand. BW + stand. BW^2^ + SVL	6	**0.0**	10.2
Stand. BW + stand. BW^2^ + SVL + SVL^2^	7	5.3	3.3
Stand. BW + SVL + SVL^2^	6	3.0	1.2
Stand. ST	4	34.1	13.3
Stand. ST + stand. ST^2^	5	35.8	15.5
Stand. ST + SVL	5	16.6	10.3
Stand. ST + stand. ST^2^ + SVL	6	18.5	11.9
Stand. ST + stand. ST^2^ + SVL + SVL^2^	7	20.3	3.6
Stand. ST + SVL + SVL^2^	6	18.4	2.3

*Notes*

The best model for each reproductive measure (∆AICc = 0) is highlighted in bold.

SVL: snout‐vent length; stand.BW: standardized belly width; stand.ST: standardized stripe thickness; *k*: number of model parameters.

**Figure 3:**
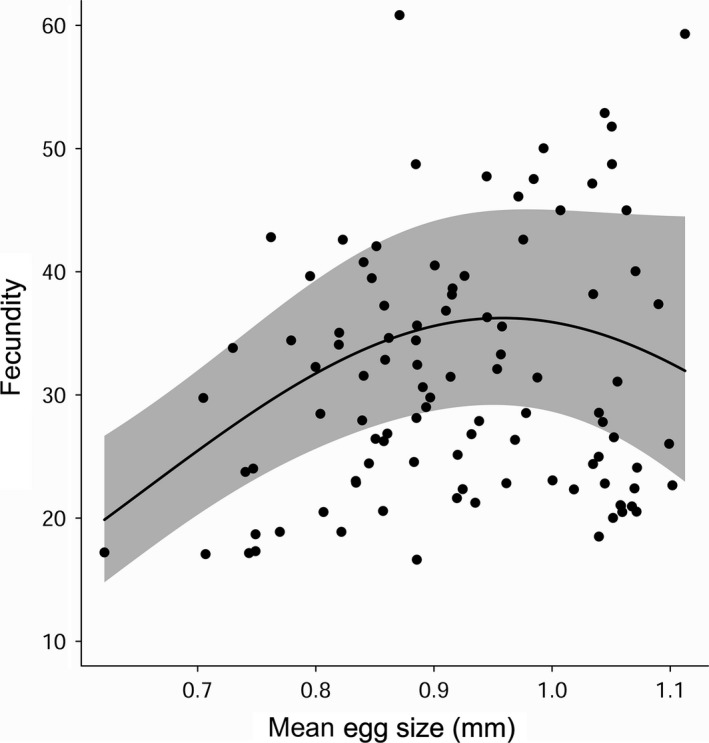
Predicted relationship (grey areas are ±95% CI) between fecundity and mean egg size, accounting for snout‐vent length (SVL). Points represent partial effects from multiple mixed‐model regression (SVL held at its mean value)

The best fecundity‐egg size model included a linear term for SVL and a quadratic term for egg size (Table 3). After accounting for larger females having higher fecundity, we found that fecundity was curve linearly related to mean egg size (*β*_(egg)_ = 0.972 [95% CI 0.436–1.490], *β*_(egg2)_ = −5.278 [95% CI −9.173 to −1.469]). Females of intermediate fecundity had the largest eggs (Figure 3).

**Table 3:** Results of AICc‐based model selection for female fecundity and mean egg size relationship

Model	*k*	∆AICc
Null	3	32.1
SVL	4	17.1
SVL + SVL^2^	5	19.2
Egg size	4	14.3
Egg size + Egg size^2^	5	6.6
SVL + Egg size	5	4.6
SVL + Egg size + Egg size^2^	6	**0**
SVL + SVL^2^ + Egg size	6	6.6
SVL + SVL^2^ + Egg size + Egg size^2^	7	1.2

*Notes*

The best model (∆AICc = 0) is highlighted in bold.

SVL: snout‐vent length; *k*: number of model parameters.

## 4 DISCUSSION

In this study, we explored the potential relationship between female ornament expression and fecundity in a species of pipefish where females possess a highly exaggerated belly ornament not found in males. We uncovered strong evidence for a positive linear relationship between ornament expression and fecundity, with larger females having proportionately larger ornaments and higher fecundity than their smaller counterparts. Ornaments often scale by traits such as body mass and/or size and are often condition dependent (Amundsen, Forsgren, & Hansen, 1997; Johnsen, Hengeveld, Blank, Yasukawa, & Nolan, 1996; Kotiaho, 2000; Simmons & Emlen, 2008). In such cases, larger or better condition females should have proportionately larger ornaments. Based on our findings, we can conclude fecundity costs to ornament expression in *S. nigra* are negligible. Instead, body size, as well as belly width and stripe thickness accurately reflect female fecundity. These results add to a growing body of literature that have similarly found no evidence of fecundity costs in species with female‐specific ornamentation (Gladbach et al., 2010; Hopkins et al., 2015; LeBas et al., 2003; Lehikoinen et al., 2010; Simmons & Emlen, 2008; Weiss, 2006; Weiss et al., 2009). Together, they challenge traditional assumptions about fecundity costs as an explanation for the scarcity of female ornament expression.

We also investigated the relationship between female body size and egg size and found that, independent of female ornamentation, larger females tended to produce smaller eggs than average size females. Such a relationship may allow larger females to produce more eggs than smaller females, but, in so doing, may also result in fitness costs to offspring. Indeed, egg size is often related to offspring fitness in a wide range of taxa, with larger eggs bestowing higher fitness benefits to developing young (Green, 2008; Krist, 2011; Weiss et al., 2009). This also appears to be the case in pipefishes (Ahnesjö, 1992), although females can also strategically mate or distribute resources to eggs and clutches depending on male quality (Braga Goncalves et al., 2010; Mobley et al., 2011; Paczolt & Jones, 2010). One potential explanation for the egg size relationships observed in our study is that large female *S. nigra* may have fewer resources to provision future broods and, hence, egg size may decrease as a result of resource depletion (Wiklund & Karlsson, 1984). However, it is currently unknown if *S. nigra* are capital breeders (i.e., pay for reproduction based on stored resources, sensu Stephens, Boyd, McNamara, & Houston, (2009) or if larger females are more successful at breeding. Alternatively, larger females may strategically decrease their egg size to increase fecundity (Parker & Begon, 1986; Smith & Fretwell, 1974). Reduction in egg and/or clutch size is hypothesized to be adaptive if the cost of reproduction declines with increasing age and if age‐selective mortality is low relative to reproduction‐dependent mortality (Begon & Parker, 1986). Finally, it is worth noting that all females were collected in the wild and may have recently mated, potentially affecting the fecundity and egg size of particular females. Currently, the ovarian type in *S. nigra* is unknown, although at least two types have thus far been described in pipefishes: the so‐called asynchronous type, where females produce small numbers of eggs continuously (e.g., *Syngnathus scovelli* and *Syngnathus typhle*: Begovac & Wallace, 1987), and the group‐synchronous type (e.g., *Nerophis ophidion*: Sogabe & Ahnesjö, 2011; *Corythoichthys haematopterus*: Sogabe et al., 2008), where ovaries mature in distinct clutches. Because mature egg size was deduced on the relative size of eggs within the ovaries based on the size of eggs within males, it is possible that we overestimated fecundity by including some nonmature eggs. Thus, if larger females recently mated and have a high proportion of nonmature eggs in the ovaries, this may account for the reduced mean egg size in the largest females.

If possessing female‐specific ornaments is not costly in terms of future offspring production, this then begs the question, why do we not see female ornaments evolve in more systems? High costs to fecundity from ornamentation may still exist in species that do not have appreciable female‐specific ornamentation, thus precluding the evolution of such structures in the first place. Moreover, if body size is the best predictor of fecundity, then additional ornaments may be superfluous (Hopkins et al., 2015), especially if they are costly in other ways (e.g., increased risk of predation). Among species that do possess female‐specific ornaments, increased signal efficacy and/or condition dependence may help to explain why female ornaments are maintained when no clear cost to fecundity is apparent. For example, larger individuals may be more effective at advertising and, as a result, signal strength may increase with body size (Tazzyman et al., 2013, 2014). Alternatively, if condition (i.e., fecundity) increases with body size, then we would expect to see larger females honestly advertising their condition (Tazzyman et al., 2014). It is interesting to note that the stripe pattern of *S. nigra*'s ornament is common in pipefishes and may accentuate body depth via the so‐called Helmholtz illusion (Berglund, 2000; Rosenqvist & Berglund, 2011). This optical striped illusion creates the appearance that the belly is wider than it is and, therefore, may increase advertisement in larger individuals without incurring costs to production.

One underexplored hypothesis for the evolution of female ornamentation is their use in female–female competition and social signaling (Lyon & Montgomerie, 2012; Rosenqvist, 1990; Rosvall, 2011; Tobias et al., 2012; West‐Eberhard, 1979). Currently, little is known if the ornament in *S. nigra* is used primarily to signal to males during courtship or in social interactions between females, such as suppressing display times of rival females. For example, in the sex‐role reversed broad snouted pipefish, *S. typhle*, the visual ornaments displayed on the flanks of females play an important role in female–female competition by functioning as a “badge of status” to intimidate rivals (Berglund & Rosenqvist, 2009). If female ornamentation is strongly influenced by social signaling, this may help to maintain honest signaling in the face of negligible costs to fecundity. Future studies could manipulate social context (e.g., interactions between rivals vs. potential mates) to elucidate the nature of the female ornament in this species.

Another possible explanation for the origins of the female ornament is that it may have evolved through sensory bias. Studies of sexual selection have shown that mate preference for sexual ornaments can sometimes arise from preexisting perceptual biases (Endler, 1992; Kirkpatrick & Ryan, 1991; Kokko, Brooks, Jennions, & Morley, 2003; Macías Garcia & Ramirez, 2005; Ryan, 1998) that reflect ecological constraints (Endler, 1992; Proctor, 1992) or basic properties of nervous systems (Rosenthal & Evans, 1998; Ryan, 1998; Ryan & Keddy‐Hector, 1992). A classic example of a preexisting female preference for a sexually selected trait is found among fishes in the genus *Xiphophorus* (Basolo, 1990, 1995). Here, female preference for the male “sword” ornament, a colorful extension of the male's caudal fin, appears to have arisen from a general female preference for larger males (Rosenthal & Evans, 1998). In goodeiid fishes, Macías Garcia and Ramirez (2005) demonstrated how such biases, in turn, can evolve into honest sexual signals. Such a possibility in the context of male preferences for body width and stripe pattern of female *S. nigra* warrants further investigation.

To conclude, we found no evidence of a fecundity cost associated with the expression of an extravagant female ornament in a sex role‐reversed pipefish, although a potential trade‐off between fecundity and egg size was uncovered. Our study adds to a growing body of empirical studies that question whether theoretical assumptions concerning the cost of female ornamentation to fecundity is warranted. Future studies should investigate alternative explanations for the evolution of female ornamentation in this and other species in nature including signaling efficacy, condition dependence, social selection, and sensory bias.

## COMPETING INTEREST

We declare we have no competing and financial interests.

## AUTHORS’ CONTRIBUTIONS

MW and BBMW initiated the study. MW performed all dissections with the assistance of DB. KBM, JRM, and MW analyzed the data. KBM drafted manuscript with help from JRM, MW, DB, BBMW. All authors gave final approval for publication.

## DATA ACCESSIBILITY

Data for this study is available from the Dryad Digital Repository: https://doi.org/10.5061/dryad.b0jt8h3

## References

[ece34459-bib-0001] Ahnesjö, I. (1992). Consequences of male brood care: Weight and number of newborn in a sex‐role reversed pipefish. Functional Ecology, 6, 274–281. 10.2307/2389517

[ece34459-bib-0002] Amundsen, T. (2000a). Female ornaments: Genetically correlated or sexually selected? In Espmark Y., Amundsen T., & Rosenqvist G. (Eds.), Animal signals: Signalling and signal design in animal communication (pp. 133–154). Trondheim, Norway: Tapir Academic Press.

[ece34459-bib-0003] Amundsen, T. (2000b). Why are female birds ornamented? Trends in Ecology & Evolution, 15, 149–155. 10.1016/S0169-5347(99)01800-5 10717684

[ece34459-bib-0004] Amundsen, T. , & Forsgren, E. (2001). Male mate choice selects for female coloration in a fish. Proceedings of the National Academy of Sciences of the United States of America, 98(23), 13155–13160. 10.1073/pnas.211439298 11606720 PMC60840

[ece34459-bib-0005] Amundsen, T. , Forsgren, E. , & Hansen, L. T. T. (1997). On the function of female ornaments: Male bluethroats prefer colourful females. Proceedings of the Royal Society B, 264, 1579–1586. 10.1098/rspb.1997.0220

[ece34459-bib-0006] Andersson, M. (1994). Sexual selection. Princeton, NJ: Princeton University Press.

[ece34459-bib-0007] Baldwin, J. , & Johnsen, S. (2012). The male blue crab, *Callinectes sapidus*, uses both chromatic and achromatic cues during mate choice. The Journal of Experimental Biology, 215(7), 1184–1191. 10.1242/jeb.067512 22399664

[ece34459-bib-0008] Barneche, D. R. , Robertson, D. R. , White, C. R. , & Marshall, D. J. (2018). Fish reproductive‐energy output increases disproportionately with body size. Science, 360(6389), 642 10.1126/science.aao6868 29748282

[ece34459-bib-0009] Basolo, A. L. (1990). Female preference predates the evolution of the sword in swordtail fish. Science, 250(4982), 808 10.1126/science.250.4982.808 17759973

[ece34459-bib-0010] Basolo, A. L. (1995). Phylogenetic evidence for the role of a pre‐existing bias in sexual selection. Proceedings of the Royal Society of London. Series B: Biological Sciences, 259(1356), 307 10.1098/rspb.1995.0045 7740048

[ece34459-bib-0011] Bates, D. , Mächler, M. , Bolker, B. , & Walker, S. (2015). Fitting linear mixed‐effects models using lme4. Journal of Statistical Software, 67(1), 1–48. https://doi.org/10.18637/jss.v067.i01

[ece34459-bib-0012] Begon, M. , & Parker, G. A. (1986). Should egg size and clutch size decrease with age? Oikos, 47, 293–302. 10.2307/3565440

[ece34459-bib-0013] Begovac, P. C. , & Wallace, R. A. (1987). Ovary of the pipefish, *Syngnathus scovelli* . Journal of Morphology, 193(2), 117–133. 10.1002/jmor.1051930202 29921108

[ece34459-bib-0014] Berglund, A. (2000). Sex role reversal in a pipefish: Female ornaments as amplifying handicaps. Annales Zoologici Fennici, 37(1), 1–13.

[ece34459-bib-0015] Berglund, A. , & Rosenqvist, G. (2009). An intimidating ornament in a female pipefish. Behavioral Ecology, 144, 54–59. 10.1093/beheco/arn114

[ece34459-bib-0016] Berglund, A. , Rosenqvist, G. , & Svensson, I. (1986a). Mate choice, fecundity and sexual dimorphism in two pipefish species (Syngnathidae). Behavioral Ecology and Sociobiology, 19(4), 301–307. 10.1007/BF00300646

[ece34459-bib-0017] Berglund, A. , Rosenqvist, G. , & Svensson, I. (1986b). Reversed sex‐roles and parental energy investment in zygotes of two pipefish (Syngnathidae) species. Marine Ecology‐Progress Series, 29(3), 209–215. 10.3354/meps029209

[ece34459-bib-0018] Bernet, P. , Rosenqvist, G. , & Berglund, A. (1998). Female‐female competition affects female ornamentation in the sex‐role reversed pipefish *Syngnathus typhle* . Behaviour, 135, 535–550. 10.1163/156853998792897923

[ece34459-bib-0019] Braga Goncalves, I. , Cornetti, L. , Couperus, A. S. , van Damme, C. J. G. , & Mobley, K. B. (2017). Phylogeography of the snake pipefish, *Entelurus aequoreus*, in the northeastern Atlantic Ocean. Biological Journal of the Linnean Society, 122, 787–800. 10.1093/biolinnean/blx112

[ece34459-bib-0020] Braga Goncalves, I. , Mobley, K. B. , Ahnesjö, I. , Sagebakken, G. , Jones, A. G. , & Kvarnemo, C. (2010). Reproductive compensation in broad‐nosed pipefish females. Proceedings of the Royal Society B, 277, 1581–1587. 10.1098/rspb.2009.2290 20106851 PMC2871843

[ece34459-bib-0021] Burnham, K. P. , & Anderson, D. R. (2002). Model selection and inference: A practical information‐theoretic approach, 2nd ed New York, NY: Springer‐Verlag.

[ece34459-bib-0022] Bussiere, L. F. , Gwynne, D. T. , & Brooks, R. (2008). Contrasting sexual selection on males and females in a role‐reversed swarming dance fly, *Rhamphomyia longicauda* Loew (Diptera: Empididae). Journal of Evolutionary Biology, 21, 1683–1691. 10.1111/j.1420-9101.2008.01580.x 18643861

[ece34459-bib-0023] Chenoweth, S. F. , Doughty, P. , & Kokko, H. (2006). Can non‐directional male mating preferences facilitate honest female ornamentation? Ecology Letters, 9(2), 179–184. 10.1111/j.1461-0248.2005.00861.x 16958883

[ece34459-bib-0024] Clutton‐Brock, T. H. (2007). Sexual selection in males and females. Science, 318, 1882–1885. 10.1126/science.1133311 18096798

[ece34459-bib-0025] Clutton‐Brock, T. H. (2009). Sexual selection in females. Animal Behaviour, 77, 3–11. 10.1016/j.anbehav.2008.08.026

[ece34459-bib-0026] Dawson, C. E. (1985). Indo‐Pacific pipefishes. Ocean Springs, MS: Gulf Coast Research Lab.

[ece34459-bib-0027] Duque‐Portugal, F. J. (1989). Distribution, growth and reproductive ecology of three pipefish from seagrass beds. MSc, University of Sidney, Sidney.

[ece34459-bib-0028] Endler, J. A. (1992). Signals, signal conditions, and the direction of evolution. The American Naturalist, 139, S125–S153. 10.1086/285308

[ece34459-bib-0029] Fitzpatrick, S. , Berglund, A. , & Rosenqvist, G. (1995). Ornaments or offspring: Cost to reproductive success restrict sexual selection processes. Biological Journal of the Linnean Society, 55, 251–260. 10.1111/j.1095-8312.1995.tb01063.x

[ece34459-bib-0030] Gladbach, A. , Gladbach, D. J. , Kempenaers, B. , & Quillfeldt, P. (2010). Female‐specific colouration, carotenoids and reproductive investment in a dichromatic species, the upland goose *Chloephaga picta leucoptera* . Behavioral Ecology and Sociobiology, 64(11), 1779–1789. 10.1007/s00265-010-0990-4 20976290 PMC2952766

[ece34459-bib-0031] Grafen, A. (1990). Biological signals as handicaps. Journal of Theoretical Biology, 144, 517–546. 10.1016/S0022-5193(05)80088-8 2402153

[ece34459-bib-0032] Green, B. S. (2008). Maternal effects in fish populations. Advances in Marine Biology, 54, 1–105. 10.1016/S0065-2881(08)00001-1 18929063

[ece34459-bib-0033] Haresign, T. W. , & Shumway, S. E. (1981). Permeability of the marsupium of the pipefish *Syngnathus fuscus* to [^14^C] alpha amino isobutyric acid. Comparative Biochemistry and Physiology A—Physiology, 69(3), 603–604. 10.1016/0300-9629(81)93030-9

[ece34459-bib-0034] Hill, G. E. (2014). The evolution of ornaments and armaments In Yasukawa K. (Ed.), Animal behavior; how and why animals do the things they do (Vol. 2: Function and Evolution, pp. 145–172). Westport, CT: Praeger.

[ece34459-bib-0035] Hopkins, J. , Baudry, G. , Candolin, U. , & Kaitala, A. (2015). I'm sexy and I glow it: Female ornamentation in a nocturnal capital breeder. Biology Letters, 11, 20150599 https://doi.org/rsbl.2015.0599 26490414 10.1098/rsbl.2015.0599 PMC4650175

[ece34459-bib-0036] Huchard, E. , Benavides, J. A. , Setchell, J. M. , Charpentier, M. J. E. , Alvergne, A. , King, A. J. , … Raymond, M. (2009). Studying shape in sexual signals: The case of primate sexual swellings. Behavioral Ecology and Sociobiology, 63(8), 1231–1242. 10.1007/s00265-009-0748-z

[ece34459-bib-0037] Jenkins, G. P. , & Hamer, P. A. (2001). Spatial variation in the use of seagrass and unvegetated habitats by post‐settlement King George whiting (Percoidei: Sillaginidae) in relation to meiofaunal distribution and macrophyte structure. Marine Ecology—Progress Series, 224, 219–229. 10.3354/meps224219

[ece34459-bib-0038] Jenkins, G. P. , Walker‐Smith, G. K. , & Hamer, P. A. (2002). Elements of habitat complexity that influence harpacticoid copepods associated with seagrass beds in a temperate bay. Oecologia, 131, 598–605. 10.1007/s00442-002-0911-y 28547555

[ece34459-bib-0039] Johnsen, T. S. , Hengeveld, J. D. , Blank, J. L. , Yasukawa, K. , & Nolan, V. (1996). Epaulet brightness and condition in female Red‐winged Blackbirds. The Auk, 113, 356–362. 10.2307/4088902

[ece34459-bib-0040] Jones, A. G. , & Avise, J. C. (2001). Mating systems and sexual selection in male‐pregnant pipefishes and seahorses: Insights from microsatellite‐based studies of maternity. Journal of Heredity, 92(2), 150–158. 10.1093/jhered/92.2.150 11396573

[ece34459-bib-0041] Jones, A. G. , Rosenqvist, G. , Berglund, A. , Arnold, S. J. , & Avise, J. C. (2000). The Bateman gradient and the cause of sexual selection in a sex‐role‐reversed pipefish. Proceedings of the Royal Society B: Biological Sciences, 267(1444), 677–680. 10.1098/rspb.2000.1055 10821612 PMC1690589

[ece34459-bib-0042] Kirkpatrick, M. , & Ryan, M. J. (1991). The evolution of mating preferences and the paradox of the lek. Nature, 350(6313), 33–38. 10.1038/350033a0

[ece34459-bib-0043] Kodric‐Brown, A. , & Brown, J. H. (1984). Truth in advertising: The kinds of traits favored by sexual selection. The American Naturalist, 124(3), 309–323. 10.1086/284275

[ece34459-bib-0044] Kokko, H. , Brooks, R. , Jennions, M. D. , & Morley, J. (2003). The evolution of mate choice and mating biases. Proceedings of the Royal Society of London. Series B: Biological Sciences, 270(1515), 653 10.1098/rspb.2002.2235 12769467 PMC1691281

[ece34459-bib-0045] Kotiaho, J. S. (2000). Testing the assumptions of conditional handicap theory: Costs and condition dependence of a sexually selected trait. Behavioral Ecology and Sociobiology, 48, 188–194. 10.1007/s002650000221

[ece34459-bib-0046] Krist, M. (2011). Egg size and offspring quality: A meta‐analysis in birds. Biological Reviews, 86(3), 692–716. 10.1111/j.1469-185X.2010.00166.x 21070586

[ece34459-bib-0047] Kuiter, R. H. (2009). Seahorses and their relatives. Seaford, Vic.: Aquatic Photographics.

[ece34459-bib-0048] Kvarnemo, C. , & Ahnesjö, I. (1996). The dynamics of operational sex ratios and competition for mates. Trends in Ecology and Evolution, 11(10), 404–408. 10.1016/0169-5347(96)10056-2 21237898

[ece34459-bib-0049] Kvarnemo, C. , Mobley, K. B. , Partridge, C. , Jones, A. G. , & Ahnesjö, I. (2011). Evidence of paternal nutrient provisioning to embryos in the pipefish *Syngnathus typhle* . Journal of Fish Biology, 78, 1725–1737. 10.1111/j.1095-8649.2011.02989.x 21651524

[ece34459-bib-0050] LeBas, N. R. (2006). Female finery is not for males. Trends in Ecology & Evolution, 21, 170–173. 10.1016/j.tree.2006.01.007 16701080

[ece34459-bib-0051] LeBas, N. R. , Hockham, L. R. , & Ritchie, M. G. (2003). Nonlinear and correlational sexual selection on ‘honest’ female ornamentation. Proceedings of the Royal Society B‐Biological Sciences, 270(1529), 2159–2165. 10.1098/rspb.2003.2482 PMC1691484 14561280

[ece34459-bib-0052] LeBas, N. R. , & Marshall, N. J. (2000). The role of colour in signalling and male choice in the agamid lizard *Ctenophorus ornatus* . Proceedings of the Royal Society B: Biological Sciences, 267, 445–452. 10.1098/rspb.2000.1020 10737400 PMC1690562

[ece34459-bib-0053] Lehikoinen, A. , Jaatinen, K. , & Öst, M. (2010). Do female ornaments indicate quality in eider ducks? Biology Letters, 6, 225–238. 10.1098/rsbl.2009.0744 19846446 PMC2865056

[ece34459-bib-0054] Lyon, B. E. , & Montgomerie, R. (2012). Sexual selection is a form of social selection. Philosophical Transactions of the Royal Society of London Series B, 367, 2266–2273. 10.1098/rstb.2012.0012 22777015 PMC3391428

[ece34459-bib-0055] Macías Garcia, C. , & Ramirez, E. (2005). Evidence that sensory traps can evolve into honest signals. Nature, 434, 501 10.1038/nature03363 15791255

[ece34459-bib-0056] MacReadie, P. I. , Hindell, J. S. , Jenkins, G. P. , Connolly, R. M. , & Keough, M. J. (2009). Fish responses to experimental fragmentation of seagrass habitat. Conservation Biology, 23, 644–652. 10.1111/j.1523-1739.2008.01130.x 19183213

[ece34459-bib-0057] Mobley, K. B. , Kvarnemo, C. , Ahnesjö, I. , Partridge, C. , Berglund, A. , & Jones, A. G. (2011). The effect of maternal body size on embryo survivorship in the broods of pregnant male pipefish. Behavioral Ecology and Sociobiology, 65, 1169–1177. 10.1007/s00265-010-1129-3

[ece34459-bib-0058] Mobley, K. B. , Small, C. M. , Jue, N. K. , & Jones, A. G. (2010). Population structure of the dusky pipefish (*Syngnathus floridae*) from the Atlantic and Gulf of Mexico, as revealed by mitochondrial DNA and microsatellite analyses. Journal of Biogeography, 37, 1363–1377. 10.1111/j.1365-2699.2010.02288.x

[ece34459-bib-0059] Nur, N. , & Hasson, O. (1984). Phenotypic plasticity and the handicap principle. Journal of Theoretical Biology, 110, 275–297. 10.1016/S0022-5193(84)80059-4

[ece34459-bib-0060] Paczolt, K. A. , & Jones, A. G. (2010). Postcopulatory sexual selection and sexual conflict in the evolution of male pregnancy. Nature, 464, 401–404. 10.1038/nature08861 20237568

[ece34459-bib-0061] Parker, G. A. , & Begon, M. (1986). Optimal egg size and clutch size: Effects of environment and maternal phenotype. The American Naturalist, 128(4), 573–592. 10.1086/284589

[ece34459-bib-0062] Partridge, C. , Shardo, J. , & Boettcher, A. (2007). Osmoregulatory role of the brood pouch in the euryhaline Gulf pipefish, *Syngnathus scovelli* . Comparative Biochemistry and Physiology A—Molecular & Integrative Physiology, 147(2), 556–561. 10.1016/j.cbpa.2007.02.007 17398130

[ece34459-bib-0063] Proctor, H. C. (1992). Sensory exploitation and the evolution of male mating behaviour: A cladistic test using water mites (Acari: Parasitengona). Animal Behaviour, 44(4), 745–752. 10.1016/S0003-3472(05)80300-8

[ece34459-bib-0064] R Core Team (2017). R: A language and environment for statistical computing. Vienna, Austria: R Foundation for Statistical Computing Retrieved from https://www.R-project.org/

[ece34459-bib-0065] Ripley, J. L. , & Foran, C. M. (2006). Differential parental nutrient allocation in two congeneric pipefish species (Syngnathidae: *Syngnathus* spp.). Journal of Experimental Biology, 209(6), 1112–1121. 10.1242/jeb.02119 16513938

[ece34459-bib-0066] Ripley, J. L. , & Foran, C. M. (2009). Direct evidence for embryonic uptake of paternally‐derived nutrients in two pipefishes (Syngnathidae: *Syngnathus* spp.). Journal of Comparative Physiology B—Biochemistry & Molecular Biology, 179, 325–333. 10.1007/s00360-008-0316-2 19005657

[ece34459-bib-0067] Rosenqvist, G. (1990). Male mate choice and female‐female competition for mates in the pipefish *Nerophis ophidion* . Animal Behaviour, 39, 1110–1115. 10.1016/S0003-3472(05)80783-3

[ece34459-bib-0068] Rosenqvist, G. , & Berglund, A. (2011). Sexual signals and mating patterns in Syngnathidae. Journal of Fish Biology, 78, 1647–1661. 10.1111/j.1095-8649.2011.02972.x 21651521

[ece34459-bib-0069] Rosenthal, G. G. , & Evans, C. S. (1998). Female preference for swords in *Xiphophorus helleri* reflects a bias for large apparent size. Proceedings of the National Academy of Sciences of the United States of America, 95(8), 4431–4436. 10.1073/pnas.95.8.4431 9539754 PMC22506

[ece34459-bib-0070] Rosvall, K. A. (2011). Intrasexual competition in females: Evidence for sexual selection? Behavioral Ecology, 22, 1131–1140. 10.1093/beheco/arr106 22479137 PMC3199163

[ece34459-bib-0071] Roulin, A. , Ducrest, A. L. , Balloux, F. , Dijkstra, C. , & Riols, C. (2003). A female melanin ornament signals offspring fluctuating asymmetry in the barn owl. Proceedings of the Royal Society B: Biological Sciences, 267, 937–941. 10.1098/rspb.2002.2215 PMC1691231 12590755

[ece34459-bib-0072] Ryan, M. J. (1998). Sexual selection, receiver biases, and the evolution of sex differences. Science, 281(5385), 1999 10.1126/science.281.5385.1999 9748154

[ece34459-bib-0073] Ryan, M. J. , & Keddy‐Hector, A. (1992). Directional patterns of female mate choice and the role of sensory biases. The American Naturalist, 139, S4–S35. 10.1086/285303

[ece34459-bib-0074] Simmons, L. M. , & Emlen, D. J. (2008). No fecundity cost of female secondary sexual trait expression in the horned beetle *Onthophagus sagittarius* . Journal of Evolutionary Biology, 21, 1227–1235. 10.1111/j.1420-9101.2008.01575.x 18631210

[ece34459-bib-0075] Smith, C. C. , & Fretwell, S. D. (1974). The optimal balance between size and number of offspring. The American Naturalist, 108, 499–506. 10.1086/282929

[ece34459-bib-0076] Sogabe, A. , & Ahnesjö, I. (2011). The ovarian structure and mode of egg production in two polygamous pipefishes: A link to mating pattern. Journal of Fish Biology, 78, 1833–1846. 10.1111/j.1095-8649.2011.02973.x 21651531

[ece34459-bib-0077] Sogabe, A. , Matsumoto, K. , Ohashi, M. , Watanabe, A. , Takata, H. , Murakami, Y. , … Yanagisawa, Y. (2008). A monogamous pipefish has the same type of ovary as observed in monogamous seahorses. Biology Letters, 4, 362–365. 10.1098/rsbl.2008.0157 18492646 PMC2610143

[ece34459-bib-0078] Stephens, P. A. , Boyd, I. L. , McNamara, J. M. , & Houston, A. I. (2009). Capital breeding and income breeding: Their meaning, measurement, and worth. Ecology, 90(8), 2057–2067. 10.1890/08-1369.1 19739368

[ece34459-bib-0079] Takahashi, Y. , & Watanabe, M. (2011). Male mate choice based on ontogenetic colour changes of females in the damselfly *Ischnura senegalensis* . Journal of Ethology, 29(2), 293–299. 10.1007/s10164-010-0257-6

[ece34459-bib-0080] Tazzyman, S. J. , Iwasa, Y. , & Pomiankowski, A. (2013). Signaling efficacy drives the evolution of larger sexual ornaments by sexual selection. Evolution, 68(1), 216–229. 10.1111/evo.12255 24099137 PMC3920633

[ece34459-bib-0081] Tazzyman, S. J. , Iwasa, Y. , & Pomiankowski, A. (2014). The handicap process favors exaggerated, rather than reduced, sexual ornaments. Evolution, 68(9), 2534–2549. 10.1111/evo.12450 24837599 PMC4277338

[ece34459-bib-0082] Tobias, J. A. , Montgomerie, R. , & Lyon, B. E. (2012). The evolution of female ornaments and weaponry: Social selection, sexual selection and ecological competition. Philosophical Transactions of the Royal Society of London Series B, Biological Sciences, 367, 2274–2293. 10.1098/rstb.2011.0280 22777016 PMC3391421

[ece34459-bib-0083] Trivers, R. L. (1972). Parental investment and sexual selection In Campbell B. (Ed.), Sexual selection and the descent of man 1871–1971 (pp. 136–179). London, UK: Heinemann.

[ece34459-bib-0084] Vincent, A. , Ahnesjo, I. , Berglund, A. , & Rosenqvist, G. (1992). Pipefishes and seahorses: Are they all sex‐role reversed? Trends in Ecology and Evolution, 7(7), 237–241. 10.1016/0169-5347(92)90052-D 21236017

[ece34459-bib-0085] Walther, B. A. , & Clayton, D. H. (2005). Elaborate ornaments are costly to maintain: Evidence for high maintenance handicaps. Behavioral Ecology, 16(1), 89–95. 10.1093/beheco/arh135

[ece34459-bib-0086] Weiss, S. L. (2006). Female‐specific color is a signal of quality in the striped plateau lizard (*Sceloporus virgatus*). Behavioral Ecology, 17, 726–732. 10.1093/beheco/arl001

[ece34459-bib-0087] Weiss, S. L. , Kennedy, E. A. , & Bernhard, J. A. (2009). Female‐specific ornamentation predicts offspring quality in the striped plateau lizard, *Sceloporus virgatus* . Behavioral Ecology, 20(5), 1063–1071. 10.1093/beheco/arp098

[ece34459-bib-0088] West‐Eberhard, M. J. (1979). Sexual selection, social competition, and evolution. Proceedings of the American Philosophical Society, 123, 222–234.

[ece34459-bib-0089] Wheeler, J. , Gwynne, D. T. , & Bussière, L. (2012). Stabilizing sexual selection for female ornaments in a dance fly. Journal of Evolutionary Biology, 25, 1233–1242. 10.1111/j.1420-9101.2012.02522.x 22551204

[ece34459-bib-0090] Wiklund, C. , & Karlsson, B. (1984). Egg size variation in satyrid butterflies: Adaptive vs historical, “Bauplan”, and mechanistic explanations. Oikos, 43(3), 391–400. 10.2307/3544158

[ece34459-bib-0091] Wilson, A. B. , Ahnesjö, I. , Vincent, A. C. J. , & Meyer, A. (2003). The dynamics of male brooding, mating patterns, and sex roles in pipefishes and seahorses (family Syngnathidae). Evolution, 57(6), 1374–1386. 10.1111/j.0014-3820.2003.tb00345.x 12894945

[ece34459-bib-0092] Zahavi, A. (1975). Mate selection: A selection for a handicap. Journal of Theoretical Biology, 53, 205–214. 10.1016/0022-5193(75)90111-3 1195756

[ece34459-bib-0093] Zahavi, A. (1977). The cost of honesty (further remarks on the handicap principle). Journal of Theoretical Biology, 67, 603–605. 10.1016/0022-5193(77)90061-3 904334

